# The role of obesity in sarcopenia and the optimal body composition to prevent against sarcopenia and obesity

**DOI:** 10.3389/fendo.2023.1077255

**Published:** 2023-03-01

**Authors:** Chaoran Liu, Keith Yu-Kin Cheng, Xin Tong, Wing-Hoi Cheung, Simon Kwoon-Ho Chow, Sheung Wai Law, Ronald Man Yeung Wong

**Affiliations:** ^1^ Department of Orthopaedics & Traumatology, The Chinese University of Hong Kong, Hong Kong, Hong Kong SAR, China; ^2^ Department of Mechanical and Automation Engineering, The Chinese University of Hong Kong, Hong Kong, Hong Kong SAR, China

**Keywords:** muscle, fat, sarcopenic obesity, aging, body mass index, body fat percentage

## Abstract

**Background:**

Elderly people with low lean and high fat mass, are diagnosed with sarcopenic obesity (SO), and often have poor clinical outcomes. This study aimed to explore the relationship between obesity and sarcopenia, and the optimal proportion of fat and muscle for old individuals.

**Methods:**

Participants aged 60 years or above were instructed to perform bioelectrical impedance analysis to obtain the muscle and fat indicators, and handgrip strength was also performed. Sarcopenia was diagnosed according to predicted appendicular skeletal muscle mass and function. Body mass index (BMI) and body fat percentage (BF%) were used to define obesity. The association of muscle and fat indicators were analyzed by Pearson’s correlation coefficient. Pearson Chi-Square test was utilized to estimate odds ratios (OR) and 95% confidence intervals (CI) on the risk of sarcopenia according to obesity status.

**Results:**

1637 old subjects (74.8 ± 7.8 years) participated in this study. Not only fat mass, but also muscle indicators were positively correlated to BMI and body weight (p < 0.05). Absolute muscle and fat mass in different positions had positive associations (p < 0.05). Muscle mass and strength were negatively related to appendicular fat mass percentage (p < 0.05). When defined by BMI (OR = 0.69, 95% CI [0.56, 0.86]; p = 0.001), obesity was a protective factor for sarcopenia, whilst it was a risk factor when using BF% (OR = 1.38, 95% CI [1.13, 1.69]; p = 0.002) as the definition. The risk of sarcopenia reduced with the increase of BMI in both genders. It was increased with raised BF% in males but displayed a U-shaped curve for females. BF% 26.0–34.6% in old females and lower than 23.9% in old males are recommended for sarcopenia and obesity prevention.

**Conclusion:**

Skeletal muscle mass had strong positive relationship with absolute fat mass but negative associations with the percentage of appendicular fat mass. Obesity was a risk factor of sarcopenia when defined by BF% instead of BMI. The management of BF% can accurately help elderly people prevent against both sarcopenia and obesity.

## 1 Introduction

The aging population has been an important challenge in public health and is posing a huge socioeconomic burden (1). A recent cohort study indicated that increased body mass index (BMI) was associated with lower all-cause and non-cardiovascular disease mortality in Chinese old people (2). This observation supports the “obesity paradox” again. However, gaining BMI can also have undesirable metabolic risks including excess adiposity accumulation, which leads to cardiovascular diseases and diabetes mellitus (3). Body composition analyses have also reported that excess body fat increases all-cause and disease-cause mortality, and people with low lean mass have been found to have higher death rates (4, 5). Therefore, the management of an optimal body composition for old people is important. It is well known that BMI only considers body mass rather than body composition, which may not be appropriate for old individuals (2), and understanding the optimal body composition to balance fat and lean mass is warranted (6).

Four main phenotypes have been classified with body adiposity and muscle mass composition, which are sarcopenia, obesity, sarcopenic obesity, and healthy status (7). Sarcopenia is an age-related muscle disorder, and is associated with increased risk of fall, fracture, and mortality (8, 9). The Asian Working Group for Sarcopenia (AWGS) 2019 consensus recommends using lower muscle mass with poorer grip strength or physical performance to define sarcopenia (10). On the other hand, the European Working Group on Sarcopenia in Older People 2 (EWGSOP2) revised consensus identifies sarcopenia in older adults with low grip strength and muscle mass, and those with a combination of poor physical performance are considered to have severe sarcopenia (11). It is known that lower BMI is commonly found in people with sarcopenia (12). Similar to BMI, body fat mass indicators including body fat percentage (BF%), are also used to diagnose obesity and estimate the risks of obesity-related diseases in older people (13, 14). Old individuals with both low muscle mass and high adiposity are sarcopenic obese (SO) which fail to benefit from the “obesity paradox” due to their higher risk of all-cause mortality (15). There has been evidence from pre-clinical studies indicating that adipose tissue damages muscle homeostasis, resulting in muscle atrophy and regeneration capacity reduction (16, 17). This finding was regarded as the pathogenic mechanism of sarcopenic obesity (17). Since sarcopenia, obesity, and sarcopenic obesity all lead to various adverse clinical outcomes of old people, it is necessary to establish the proper body indicator cut-offs for reference to decrease relevant risks. This cross-sectional study aims to explore i) the relationship between fat and muscle indicators in Asian elderly people, ii) the role of obesity in sarcopenia and muscle maintenance based on BMI- and BF%-defined obesity, and iii) the optimal BMI and BF% to prevent against both sarcopenia and obesity in old individuals.

## 2 Materials and methods

### 2.1 Study population

Elderly people were screened from the community or outpatient clinics at Prince of Wales Hospital in Hong Kong from 2019 to 2021. The inclusion criteria were 1) aged 60 years old or above, and 2) Chinese ethnicity. The exclusion criteria were: 1) severe foot deformity which is unable to acquire the BIA data, and 2) unable to communicate and understand the test instructions, e.g., severe dementia. This study was approved by The Joint Chinese University of Hong Kong – New Territories East Cluster Clinical Research Ethics Committee (Ref. CREC 2018.602).

### 2.2 Assessment of muscle and fat

All participants height were measured by an ultrasonic sensor (Clifford H.K. Co., Hong Kong). The whole-body skeletal muscle mass (SMM), body fat mass (BFM), arms fat mass (AFM), legs fat mass (LFM), and trunk fat mass (TFM), arms fat-free mass (AFFM), legs fat-free mass (LFFM), as well as waist-hip ratio (WHR) were assessed and directly obtained from the bioelectrical impedance analysis (BIA) system (InBody 120, Seoul, Korea). The tests were performed according to the manual instructions. In brief, subjects stood on the BIA device platform barefoot, and held the electrodes until the measurement was completed. Other body composition values were calculated as follows: fat mass index (FMI) = BFM/height^2^, skeletal muscle mass index (SMI) = SMM/height^2^, BF% = BFM/body weight, leg fat mass percentage (LFM%) = LFM/leg mass, arms fat mass percentage (AFM%) = AFM/arm mass, trunk fat mass percentage (TFM%) = TFM/trunk mass, leg fat-free mass percentage (LFFM%) = LFFM/leg mass, arm fat-free mass percentage (AFFM%) = AFFM/arm mass. We previously found that the value of muscle mass index detected by BIA (InBody 120) was 2.89 ± 0.38 kg/m^2^ higher than measured by dual-energy X-ray absorptiometry (DXA) (Horizon, Hologic, Marlborough, MA, USA), which was considered the gold standard (18). Therefore, we recruited another 48 volunteers and utilized our previous method to establish a model to predict the DXA-measured appendicular skeletal muscle mass index (ASMI) based on BIA-measured SMI and demographic information *via* test- (n=32) and validation (n=16) groups (18). Multiple regression and Bland–Altman analyses were performed. SMI, age, sex, and anthropometric parameters including height, weight, and BMI were involved as potential contributions to establish the best model (18). The final prediction model is: ASMI (DXA) = 0.378 + 0.662 * (BIA SMI) – 0.003 * (Age) – 0.032 * (BMI); R^2^ = 0.862. The mean difference between predicted and actual value was 0.04 ± 0.25 kg/m^2^ in the validation group. Handgrip strength (HGS) was measured by the dynamometer (5030JI, JAMAR, Bolingbrook, IL, USA). Participants seated with 90° elbow flexion and executed the test 3 trials per hand. The maximal reading was recorded (10).

### 2.3 Diagnosis of sarcopenia and obesity

Cut-off points according to the AWGS 2019 were used. Participants with both low muscle mass and strength was defined as sarcopenia. Male with ASMI (predicted) < 7.0 kg/m^2^, and HGS < 28 kg, and female with ASMI (predicted) < 5.4 kg/m^2^, and HGS < 18 kg were sarcopenic. Two criteria were used to diagnose obesity according to the previous studies of SO (19). The BMI ≥ 25 kg/m^2^ was used to define obesity as recommended by WHO for East Asians (20); and BF% > 27% in male and 35% in female, which was used in previous SO studies for classification of obesity, and was close to the 60th percentile of BF% in our cohort (21–23).

### 2.4 Statistical analyses

Continuous variables were presented as mean ± standard error (SD), and categorical variables were expressed as number and percentage. Pearson’s correlation coefficient was used to test the correlations between variables, including age, height, weight, muscle- and fat-related indicators. One-way ANOVA with *post-hoc* analysis by Bonferroni test was used to analyze the differences of body parameters between normal, only sarcopenic, only obese, and sarcopenic obese groups. The Pearson Chi-square test was performed to detect the role of obesity in sarcopenia *via* odds ratios (OR), as well as the proper values of BMI and BF% to prevent sarcopenia according to the fifth distributions of BMI and BF%. The age-related descent rate of muscle mass and strength in people with or without obesity, as well as the ASMI prediction model were estimated by using regression coefficient (β) from linear regression analysis. Python 3.10.1 and R 4.0.2 were utilized for the analyses. p ≤ 0.05 was regarded as statistical significance in differences.

## 3 Results

### 3.1 The associations of fat and muscle indicators

**Figure 1:**
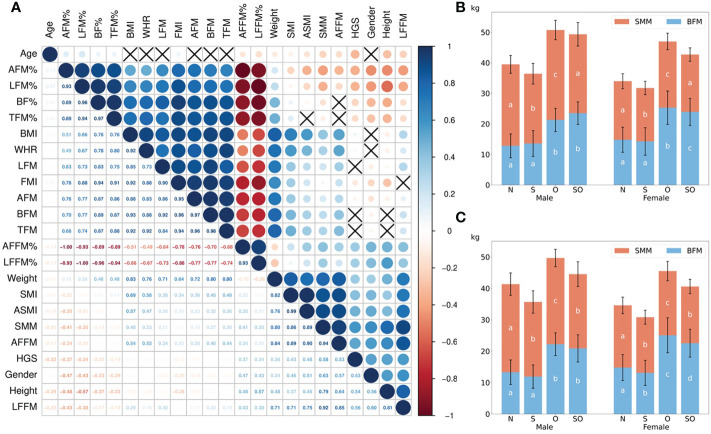
The correlation between muscle and fat indicators and the differences between normal, sarcopenic, obese, and sarcopenic obese groups. In **(A)**, the dark blue showed the strong positive correlation (correlation coefficient = 1), while the dark red showed the strong negative correlation (correlation coefficient = –1). Black cross was shown if there was no statistical significance (P > 0.05). The correlation coefficient was displayed in the lower half of the square. Female=0, male=1 for gender. **(B, C)** showed the differences of SMM and BFM in four groups according to BMI- and BF%-defined obesity. The *post-hoc* results were shown as a, b, c, d on the bars with same color; the results in groups with inconsistent letters were significantly different (P < 0.05). AFM%, arm fat mass percentage; LFM%, leg fat mass percentage; BF%, body fat percentage; TFM%, trunk fat mass percentage; BMI, body fat index; WHR, waist to hip ratio; LFM, leg fat mass; FMI, fat mass index; AFM, arm fat mass; BFM, body fat mass; TFM, trunk fat mass; AFFM%, arm fat-free mass percentage; LFFM%, leg fat-free mass percentage; SMI, skeletal muscle mass index; ASMI, appendicular skeletal muscle mass index; SMM, skeletal muscle mass; AFFM, arm fat-free mass; HGS, handgrip strength; LFFM, leg fat-free mass; N, normal group; S, only sarcopenic group; O, only obese group; SO, sarcopenic obese group.

1637 old subjects (age: 74.8 ± 7.8, range: 60–98 years; 83.6% female) were included without missing data (**Table 1**). After analyzing data from the whole cohort (both genders), the Pearson’s correlations (**Figure 1A**) showed that age (≥ 60 years) was not related to BMI, WHR, and fat mass in different body positions (p > 0.05). Higher fat mass percentage in the whole and partial body, fat mass index, and lower weight, height, fat-free mass (FFM) in partial body, percentage of FFM, SMM, SMI, ASMI, and handgrip strength were related to increased age (p < 0.05). The percentage of fat mass in arms and legs were inversely correlated with SMM, SMI, and ASMI (p < 0.05). TFM% was positively related to SMI (p < 0.001), but not ASMI (p > 0.05). Higher TFM% was associated with reduced SMM and HGS (p < 0.05). BF% was weakly and negatively related to SMM and ASMI, but positively related to SMI (p < 0.05). Body weight, BMI, absolute fat mass, and WHR had similar trends to be positively associated with almost all muscle and fat parameters instead of fat-free mass percentage (p < 0.05). ASMI and HGS were both negatively related to the percentage of fat mass in limbs (p < 0.05). The correlation of muscle and fat indicators in females and males was shown **Supplementary Figure 1**. In males, SMI and WHR reduced with advanced age, which were not significant in females. SMM in both genders was negatively related to percentage of appendicular fat mass (p < 0.05), but positively associated with BF% in females. The inverse association between TFM% and HGS was only found in males rather than females. ASMI was inversely related to AFM% but not LFM% in both genders. In females, SMI increased with higher LFM%, and HGS increased with higher WHR, which were not found in males.

**Table 1:** The prevalence, muscle and fat indicators in older people with normal status, sarcopenia, obesity, and SO.

	Obesity defined by BMI				Obesity defined by BF%			
	Normal	Sarcopenia	Obesity	SO	Normal	Sarcopenia	Obesity	SO
Male								
N (prevalence)	77(28.7%)	98(36.6%)	55(20.5%)	38(14.2%)	87(32.4%)	69(25.7%)	45(16.8%)	67(25.0%)
Age (years)	71.1 ± 4.8^a^	79.5 ± 7.5^b^	71.3 ± 5.5^a^	77.5 ± 8.8^b^	70.8 ± 4.9^a^	78.5 ± 7.9^b^	71.8 ± 5.5^a^	79.3 ± 7.9^b^
Height (cm)	166.6 ± 5.8^a^	160.3 ± 6.9^b^	165.1 ± 5.2^a^	160.6 ± 7.7^b^	167.0 ± 5.9^ab^	161.7 ± 6.7^cd^	164.0 ± 4.3^ac^	159.0 ± 7.3^d^
Weight (kg)	61.3 ± 6.5^a^	55.9 ± 7.7^b^	74.4 ± 6.3^c^	70.9 ± 8.1^c^	64.1 ± 8.9^a^	55.8 ± 8.6^b^	72.0 ± 7.2^c^	64.5 ± 10.1^a^
BMI (kg/m^2^)	22.1 ± 2.0^a^	21.7 ± 2.3^a^	27.3 ± 2.0^b^	27.4 ± 1.7^b^	23.0 ± 2.8^a^	21.3 ± 2.4^b^	26.8 ± 2.7^c^	25.4 ± 2.8^c^
BF%	20.7 ± 5.2^a^	24 ± 6.2^b^	28.6 ± 4.4^c^	33.2 ± 4.3^d^	20.5 ± 4.2^a^	21.0 ± 4.9^a^	30.8 ± 3.1^b^	32.3 ± 3.4^b^
SMI (kg/m^2^)	9.6 ± 0.8^a^	8.9 ± 0.9^b^	10.8 ± 0.9^c^	10.0 ± 0.8^a^	10.0 ± 1.0^a^	9.1 ± 0.9^b^	10.2 ± 1.0^a^	9.3 ± 1.0^b^
ASMI (kg/m^2^)	5.8 ± 0.5^a^	5.3 ± 0.5^b^	6.4 ± 0.5^c^	5.9 ± 0.5^a^	6.1 ± 0.6^a^	5.5 ± 0.6^b^	6.1 ± 0.6^a^	5.5 ± 0.6^b^
HGS (kg)	31.7 ± 2.8^a^	20.9 ± 4.9^b^	31.6 ± 4.5^a^	20.6 ± 4.5^b^	32.0 ± 3.8^a^	21.7 ± 4.6^b^	31.1 ± 3.0^a^	19.9 ± 4.8^b^
FMI (kg/m^2^)	4.6 ± 1.4^a^	5.3 ± 1.7^b^	7.8 ± 1.6^c^	9.1 ± 1.5^d^	4.8 ± 1.4^a^	4.5 ± 1.4^a^	8.3 ± 1.4^b^	8.3 ± 1.6^b^
WHR	0.83 ± 0.04^a^	0.83 ± 0.04^a^	0.90 ± 0.03^b^	0.90 ± 0.03^b^	0.84 ± 0.04^a^	0.82 ± 0.04^b^	0.90 ± 0.03^c^	0.89 ± 0.03^c^
AFFM (kg)	4.9 ± 0.8^a^	4.1 ± 0.9^b^	5.9 ± 0.9^c^	4.9 ± 1.0^a^	5.3 ± 1.0^a^	4.2 ± 1.0^b^	5.4 ± 0.8^a^	4.4 ± 1.0^b^
LFFM (kg)	15.1 ± 1.9^a^	12.5 ± 2.3^b^	16.3 ± 1.7^c^	14.3 ± 2.5^a^	15.7 ± 2.1^a^	13.1 ± 2.4^b^	15.3 ± 1.5^a^	12.9 ± 2.7^b^
AFM (kg)	1.5 ± 0.6^a^	1.8 ± 0.6^a^	2.8 ± 0.8^b^	3.4 ± 0.7^c^	1.5 ± 0.5^a^	1.5 ± 0.5^a^	3.0 ± 0.7^b^	2.9 ± 0.8^b^
LFM (kg)	4.1 ± 1.1^a^	4.3 ± 1.2^a^	6.3 ± 1.2^b^	7.1 ± 1.3^c^	4.2 ± 1.0^a^	3.8 ± 1.0^a^	6.7 ± 1.1^b^	6.4 ± 1.4^b^
TF (kg)	6.2 ± 2.2^a^	6.6 ± 2.4^a^	11.0 ± 2.0^b^	11.8 ± 1.9^b^	6.5 ± 2.4^a^	5.6 ± 2.2^a^	11.4 ± 1.9^b^	10.5 ± 2.3^b^
AFFM%	76.5 ± 7.2^a^	70.1 ± 8.3^b^	68.1 ± 7.2^b^	59.3 ± 7.2^c^	77.6 ± 5.3^a^	74.0 ± 6.4^b^	64.1 ± 5.1^c^	60.0 ± 6.0^d^
LFFM%	78.8 ± 4.7^a^	74.6 ± 6.0^b^	72.1 ± 4.3^c^	66.6 ± 5.0^d^	79.1 ± 3.8^a^	77.6 ± 4.6^a^	70.0 ± 2.9^b^	67.0 ± 3.9^c^
AFM%	23.5 ± 7.2^a^	29.9 ± 8.3^b^	31.9 ± 7.2^b^	40.7 ± 7.2^c^	22.4 ± 5.3^a^	26.0 ± 6.4^b^	35.9 ± 5.1^c^	40.0 ± 6.0^d^
LFM%	21.2 ± 4.7^a^	25.40 ± 6.0^b^	27.9 ± 4.3^c^	33.4 ± 5.0^d^	20.9 ± 3.8^a^	22.4 ± 4.6^a^	30.0 ± 2.9^b^	33.0 ± 3.9^c^
TFM%	22.6 ± 6.4^a^	26.1 ± 7.5^b^	31.9 ± 4.3^c^	36.9 ± 4.1^d^	22.6 ± 5.6^a^	22.7 ± 6.3^a^	34.0 ± 3.0^b^	35.7 ± 3.6^b^
Female								
N (prevalence)	518(37.8%)	424(31.0%)	265(19.4%)	162(11.8%)	512(37.4%)	349(25.5%)	271(19.8%)	237(17.3%)
Age (years)	72.1 ± 6.4^a^	76.8 ± 8.6^b^	73.8 ± 7.0^c^	79.3 ± 7.5^d^	72.4 ± 6.6^a^	76.7 ± 8.6^b^	73.2 ± 6.7^a^	78.7 ± 7.8^c^
Height (cm)	153.5 ± 6^a^	150.1 ± 6.2^b^	151.5 ± 6.0^c^	147.6 ± 6.3^d^	153.7 ± 5.8^a^	150.3 ± 6.3^b^	151.1 ± 6.1^b^	148.1 ± 6.2^c^
Weight (kg)	50.9 ± 6.4^a^	47.6 ± 6.7^b^	65.6 ± 8.3^c^	59.5 ± 6.4^d^	51.9 ± 7.4^a^	46.8 ± 6.8^b^	63.3 ± 10.0^c^	56.8 ± 7.1^d^
BMI (kg/m^2^)	21.6 ± 2.2^a^	21.1 ± 2.6^b^	28.6 ± 3.1^c^	27.3 ± 2.2^d^	21.9 ± 2.7^a^	20.7 ± 2.6^b^	27.7 ± 3.8^c^	25.9 ± 2.8^d^
BF%	28.7 ± 6.1^a^	29.3 ± 6.6^a^	38.3 ± 4.7^b^	40.1 ± 4.3^c^	28.1 ± 5.4^a^	27.4 ± 5.7^a^	39.4 ± 3.5^b^	39.5 ± 3.8^b^
SMI (kg/m^2^)	8.1 ± 0.7^a^	7.7 ± 0.7^b^	9.4 ± 0.8^c^	8.6 ± 0.5^d^	8.4 ± 0.8^a^	7.8 ± 0.7^b^	8.9 ± 1.0^c^	8.2 ± 0.7^a^
ASMI (kg/m^2^)	4.8 ± 0.4^a^	4.6 ± 0.4^b^	5.5 ± 0.5^c^	5.0 ± 0.3^d^	5.0 ± 0.5^a^	4.7 ± 0.4^b^	5.2 ± 0.6^c^	4.7 ± 0.4^b^
HGS (kg)	20.7 ± 2.8^a^	13.9 ± 3.4^b^	19.7 ± 4.4^c^	13.9 ± 3.1^b^	20.5 ± 3.1^a^	13.8 ± 3.4^b^	20.1 ± 3.9^a^	13.9 ± 3.1^b^
FMI (kg/m^2^)	6.3 ± 1.8^a^	6.3 ± 2.0^a^	11.0 ± 2.5^b^	11.0 ± 2.0^b^	6.3 ± 1.7^a^	5.8 ± 1.7^b^	11.0 ± 2.5^c^	10.3 ± 2.0^d^
WHR	0.83 ± 0.04^a^	0.82 ± 0.05^b^	0.93 ± 0.04^c^	0.92 ± 0.04^d^	0.84 ± 0.05^a^	0.82 ± 0.05^b^	0.92 ± 0.05^c^	0.90 ± 0.04^d^
AFFM (kg)	3.2 ± 0.6^a^	2.8 ± 0.6^b^	4.1 ± 0.7^c^	3.4 ± 0.7^d^	3.4 ± 0.7^a^	2.8 ± 0.6^b^	3.8 ± 0.8^c^	3.2 ± 0.7^d^
LFFM (kg)	10.2 ± 1.7^a^	9.0 ± 1.6^a^	11.3 ± 1.8^b^	9.7 ± 2.8^c^	10.5 ± 1.8^a^	9.2 ± 1.6^b^	10.7 ± 2^a^	9.3 ± 2.5^b^
AFM (kg)	2.0 ± 0.6^a^	2.0 ± 0.7^a^	3.8 ± 1.2^b^	3.6 ± 1.0^c^	2.0 ± 0.6^a^	1.8 ± 0.5^b^	3.8 ± 1.2^c^	3.4 ± 0.9^d^
LFM (kg)	4.7 ± 1.2^a^	4.6 ± 1.3^a^	7.6 ± 1.8^b^	7.5 ± 2.7^b^	4.7 ± 1.2^a^	4.2 ± 1.1^b^	7.6 ± 1.8^c^	7.0 ± 2.4^d^
TF (kg)	7.1 ± 2.3^a^	6.7 ± 2.6^a^	12.7 ± 2.6^b^	11.8 ± 2.4^c^	7.1 ± 2.4^a^	6.1 ± 2.3^b^	12.5 ± 2.7^c^	11.1 ± 2.4^d^
AFFM%	61.5 ± 7.8^a^	58.7 ± 8.0^b^	52.3 ± 6.8^c^	48.9 ± 6.9^d^	62.8 ± 6.6^a^	61.1 ± 6.7^b^	50.0 ± 5.0^c^	48.5 ± 5.9^d^
LFFM%	68.5 ± 6.0^a^	66.7 ± 6.6^b^	59.8 ± 5.0^c^	56.7 ± 4.7^d^	69.3 ± 5.2^a^	68.6 ± 5.6^a^	58.6 ± 4.0^b^	57.1 ± 4.2^c^
AFM%	38.5 ± 7.8^a^	41.4 ± 8.0^b^	47.7 ± 6.8^c^	51.1 ± 6.9^d^	37.2 ± 6.6^a^	39.0 ± 6.6^b^	50.0 ± 5.0^c^	51.5 ± 5.9^d^
LFM%	31.5 ± 6.0^a^	33.3 ± 6.6^b^	40.2 ± 5.0^c^	43.3 ± 4.7^d^	30.7 ± 5.2^a^	31.4 ± 5.6^a^	41.4 ± 4.0^b^	42.9 ± 4.2^c^
TFM%	31.0 ± 7.3^a^	31.6 ± 8.1^a^	41.5 ± 4.3^b^	43.1 ± 5.0^b^	30.4 ± 6.7^a^	29.4 ± 7.3^a^	42.5 ± 3.3^b^	42.6 ± 4.3^b^

a, b, c, d: variables in groups with different letters were significantly different (p < 0.05).

### 3.2 The characteristics of sarcopenia, obesity, and sarcopenic obesity in Asian old people

Subjects were divided into four groups based on sarcopenia and two obesity definitions (**Table 1**). More SO patients were detected when obesity was defined by BF% (25% in male, 17.3% in female, and 18.6% in total). If BMI ≥ 25 kg/m^2^ was used to define obesity, the prevalence of SO was 14.2% in male, 11.8% in female, and 12.2% in total. Fat mass percentage in the trunk was similar between individuals with sarcopenia and non-sarcopenia when compared within the people with or without obesity, respectively (p > 0.05), except for males defined with obesity by BMI. WHR was similar or higher in the healthy group compared to only sarcopenic group, as well as in only obese group compared to sarcopenic obesity group. Appendicular fat mass was comparable or lower in sarcopenic groups with matched obesity status, but significantly higher when demonstrated by percentage. The highest percentage of arm and leg fat mass was found in SO (p < 0.05). Although BFM was similar between obese status-matched sarcopenic and non-sarcopenic groups, lower SMM was shown in the former groups (**Figures 1B, C**). With similar ASMI, BMI-defined SO had remarkedly higher BF% and lower HGS than the normal group (p < 0.05). There were no significant differences of ASMI and HGS between the two sarcopenic groups when obesity was defined by BF% (p > 0.05).

### 3.3 The role of obesity in sarcopenia and muscle maintenance

**Figure 2:**
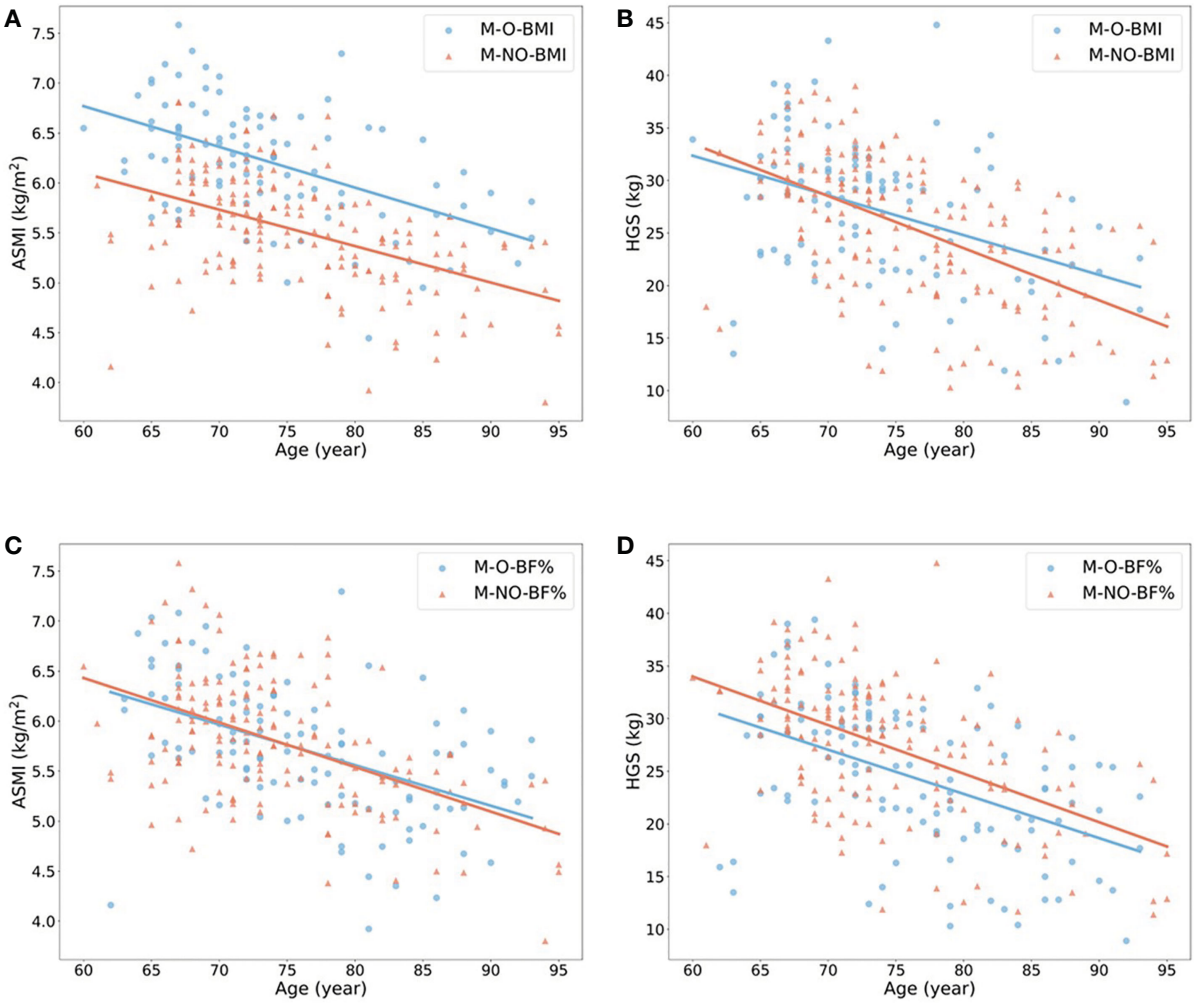
Linear regression model to show the annual rate of ASMI and HGS decline in females with (blue) or without (red) obesity. **(A, B)** showed the changes of ASMI and HGS according to age in females with or without obesity when obesity defined by BMI ≥ 25 kg/m2. (**C, D)** showed the changes of the above variables when obesity defined by body fat percentage > 35% in female. All p-value of regression models is ≤ 0.05. F, female; O, obese; NO, non-obese; BMI, body mass index; BF%, body fat percentage; ASMI, appendicular skeletal muscle mass index; HGS, handgrip strength.

**Figure 3:**
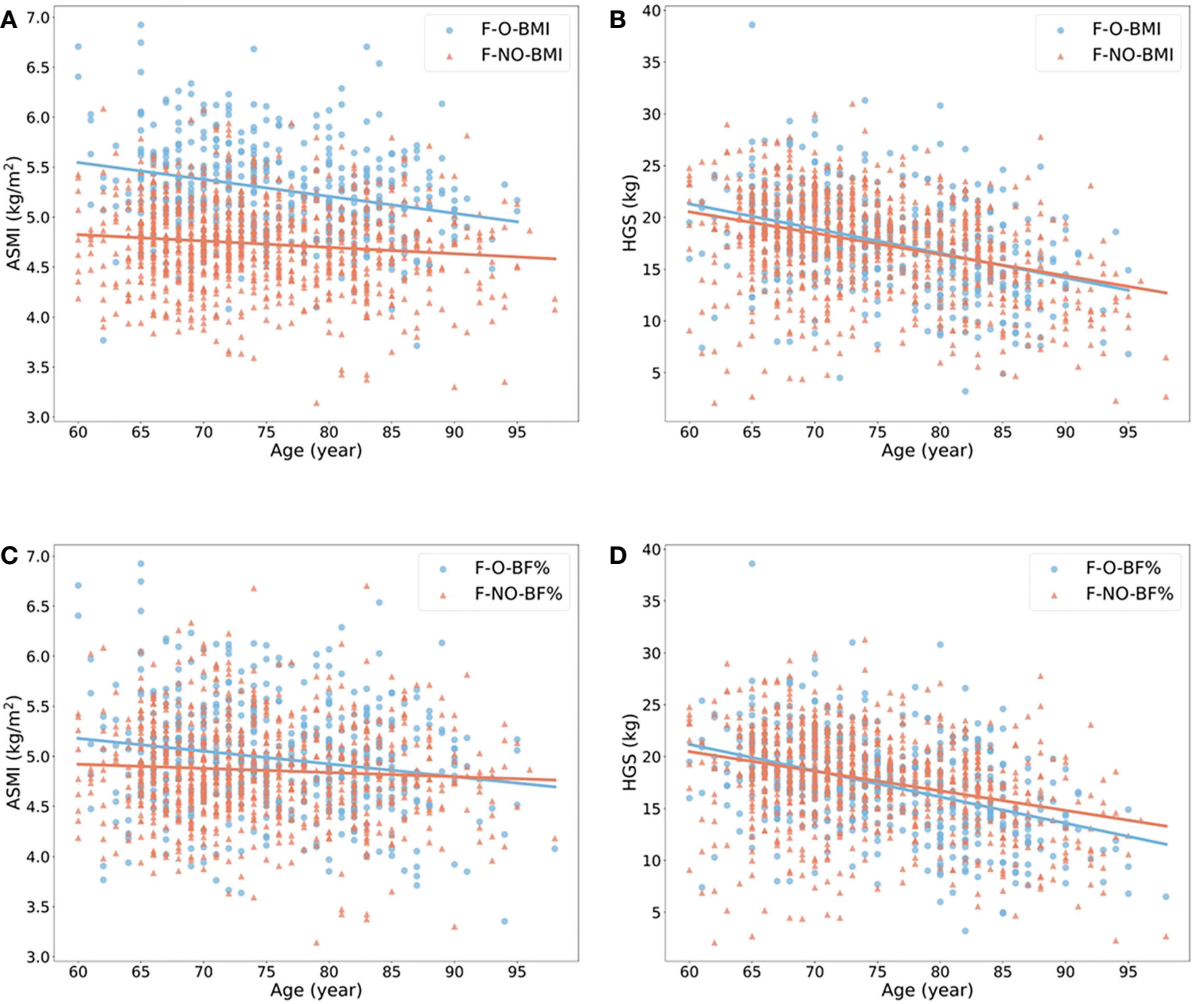
Linear regression model to show the annual rate of ASMI and HGS decline in males with (blue) or without (red) obesity. **(A, B)** showed the changes of ASMI and HGS according to age in males with or without obesity when obesity defined by BMI ≥ 25 kg/m2. **(C, D)** showed the changes of the above variables when obesity defined by body fat percentage > 27% in male. All p-value of regression models is < 0.05. M, male; O, obese; NO, non-obese; BMI, body mass index; BF%, body fat percentage; ASMI, appendicular skeletal muscle mass index; HGS, handgrip strength.

The ORs with 95% confidence interval (CI) showed the risk of sarcopenia in elderlies with obesity (**Table 2**). BMI- and BF% defined obesity had opposite roles in sarcopenia. When the population without obesity was regarded as the reference group (OR = 1.00), obesity defined by BMI was a protective factor of sarcopenia in both male and female (ORs < 1.00, p < 0.05), while BF%-defined obesity was a risk factor (ORs > 1.00, p < 0.05). We also estimated the annual rate of muscle mass and strength decline based on obesity status in the elderly females (**Figures 2A–D**) and males (**Figures 3A–D**). For females, individuals with obesity had a steeper slope of ASMI (β: -0.017 vs. -0.006) and HGS (β: -0.238 vs. -0.206) decline when defined by BMI. Similar trends were also found in BF%-defined females with obesity, with the regression coefficient (β: -0.013 vs. -0.004) in ASMI, and in HGS (β: -0.253 vs. -0.189). Faster decline of ASMI in BMI-defined male with obesity was identified (β: -0.041 vs. -0.037). Other indicators in male without obesity declined more than male with obesity. **Supplementary Table 1** showed the corresponding regression equations.

**Table 2:** The risk of sarcopenia according to the status of obesity.

Definition	Gender	OR non-obese	OR obese	95%CI		p-value
				lower	upper	
BMI	Male	1.00 (reference)	0.53	0.32	0.88	0.013
	Female	1.00 (reference)	0.73	0.58	0.92	0.007
	Total	1.00 (reference)	0.69	0.56	0.86	0.001
BF%	Male	1.00 (reference)	1.88	1.15	3.07	0.012
	Female	1.00 (reference)	1.28	1.03	1.60	0.027
	Total	1.00 (reference)	1.38	1.13	1.69	0.002

### 3.4 Optimal BMI and BF% in the elderly to decrease risk of sarcopenia

**Figure 4:**
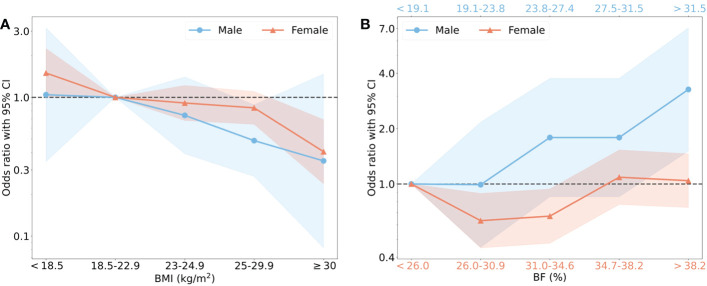
The risk of sarcopenia in males and females with different BMI and BF%. **(A)** showed that BMI was classified into 5 intervals based on the recommendation from WHO, the normal BMI (18.5–22.9) was regarded the reference group with OR=1.00. Blue points as OR values and blue shade as 95% CI represented male, and red represented female. **(B)** showed that BF% was classified into 5 intervals by quintile, the group with the lowest value of BF% was reference group. The specific interval of male (blue) was shown on the upper horizontal axis and female (red) on the lower horizontal axis.

To specify the optimal BMI and BF% that should be maintained in elderlies to prevent sarcopenia, the recommended classification of BMI (<18.5, 18.5–22.9, 23–24.9, 25–29.9, ≥30) (20), as well as the fifth distributions of BF% (<19.1, 19.1–23.8, 23.9–27.4, 27.5–31.5, >31.5 in males, <26.0, 26.0–30.9, 31.0–34.6, 34.7–38.2, >38.2 in females) were used to calculate the ORs of sarcopenic prevalence according to the intervals of BMI and BF% (**Supplementary Table 2**). BMI 18.5–22.9, and the lowest BF% (<19.1) were chosen as reference groups. With the increase of BMI, a trend of reduced risks of sarcopenia were found in both male and female (**Figure 4A**). The significant effect of sarcopenia prevention was found in BMI 25–29.5 group in male (p = 0.02), and BMI ≥ 30 in female (p = 0.001). BMI <18.5 increased the risk of sarcopenia in female (p = 0.05). BF% and the risk of sarcopenia displayed a U-shaped curve in female, but the OR was lineally raised in male over 23.8% (**Figure 4B**). The significant protective effects were found in BF% 26.0–30.9% and 31.0–34.6% groups compared to the lowest BF% group in female (p < 0.05). Nevertheless, the risk of sarcopenia was comparable in the first four BF% groups, but significantly higher in the fifth group with BF% > 31.5 (p < 0.01) in male. To minimize the risk of sarcopenia, females should keep their BMI over 18.5 kg/m^2^, as well as BF% between 26.0% and 34.6%. In males, higher BMI and BF% less than 23.9% were recommended.

## 4 Discussion

Muscle and fat are two widely studied tissues that contribute to a significant portion of our bodies. Without a large change of body composition, the increase of BMI is usually accompanied with both fat and muscle mass in adults. For old people, a lower BMI has become a predictor of sarcopenia (12). Various biomarkers for sarcopenia identification may be derived from this characteristic, such as lower triglycerides in sarcopenic patients (24). However, the gain of weight or BMI for elderly people without monitoring body composition is inadvisable, since older people have less lipid turnover and higher risks of metabolic diseases (25). A weak but significantly positive correlation between BF% and age was found in the elderly. This finding was also applicable in people from middle to old age (26). Although patients with sarcopenia have similar or even lower levels of absolute fat mass compared to non-sarcopenic people, their relative fat mass increased especially in limbs. Appendicular fat mass percentage was inversely related to ASMI and HGS when analyzed the whole cohort. Therefore, the fat deposition in limbs can be a potential diagnostic indicator of sarcopenia. Central obesity was associated with the development of metabolic complications and adverse clinical outcomes (27). We found higher TFM% was related to lower HGS in males, but to higher ASMI and SMI in females. Although WHR in non-sarcopenic individuals was also similar or higher compared to the sarcopenic ones, higher WHR in females was positively related to muscle mass and strength indicators. Previous studies also showed that females with central obesity but not males had lower prevalence of sarcopenia (28). This finding indicated there were greater adverse effects of fat accumulation and central obesity on the muscle of males. *In-vitro* studies showed that the coculture of mature adipocytes and skeletal muscle progenitor cells led to a reduction of nuclei number in myosin heavy chain (MHC)-positive myotubes (29). Fat deposition in extremities may play a role of muscle loss and dysfunction in sarcopenic patients *via* paracrine of adipokines and cytokines. Circulation lipid metabolites may also play roles in aggravating muscle metabolism disorders, which mainly affects the energy metabolism and muscle function (30).

There is a well-known paradox that obesity is related to a lower risk of mortality (31). However, this finding depends on the definition of obesity by using BMI. When obesity was defined by BF%, obesity became related to higher death rate (14). Hence, the body composition may be the missing gap. According to the body composition, old individuals can be separated into sarcopenia, obesity, SO, and healthy status. Individuals with SO had lower muscle mass, strength, and higher adiposity, as well as higher all-cause mortality and worse surgical prognosis (17, 32). In our study, SO was more prevalent in males than females, and when obesity was defined by BF% than BMI. When defined by BMI, SO could be diagnosed dominantly by muscle function test since their muscle mass was large. Although with higher BMI and absolute muscle mass than sarcopenia alone, SO patients had lower muscle quality, high risk of physical disability, as well as more metabolic issues, which may induce poor clinical outcomes (33). If defined by BF%, ASMI became comparable between simple sarcopenic and SO patients due to the shrunken discrepancies of BMI among groups. AFM% and LFM% were significantly higher in SO and may be biomarkers of this disease. In most cases, SO patients had different demographic features when diagnosed by different obesity definitions. A recommendation of the standard diagnostic criteria of SO should be noted in the future according to the risk of adverse events and outcomes with different definitions.

When obesity was defined by BMI, we found that it was a protective factor of sarcopenia despite the various metabolic problems that can occur (34). On the contrary, pre-clinical studies reported that obesity impaired muscle glucose tolerance, imbalanced protein synthesis and degradation, and oxidative stress which ultimately led to muscle atrophy, especially in old animals (16, 17). This may be caused by the severe obesity and exorbitant BF% in diet-induced obese animal models (35). In our study, we observed that obesity defined by BF% was a risk factor of sarcopenia which was consistent with pre-clinical findings. The ratio of body fat was not only associated with metabolic syndromes and adverse events but also with sarcopenia (36, 37). BF% contains the information of lean mass, fat mass, sarcopenia, and obesity, which is better than BMI that only contains body mass for elderly people. Similar to previous findings, in the elderly female group with obesity, a faster decline of muscle mass and strength with aging was observed (38). Although they had larger muscle storage, the muscle regeneration may be impaired (17). Nevertheless, the muscle decline in males was not as sensitive to obesity as in females. To explore the casual relationship between obesity and sarcopenia, a prospective study is needed. The management of body composition is important, and there are several strategies. Resistance training combined with nutrient supplementation, such as protein is preferable to maintain muscle mass (39). As for elderlies with obesity, the combination of caloric restriction (low-fat, proper high-protein diet with moderately decreased energy), as well as aerobic and resistance training have been recommended (40, 41).

In order to identify the optimal BMI and BF% to prevent sarcopenia, we divided the elderly population into 5 subgroups according to BMI and BF% distribution as previous methods (4). The differences caused by gender was apparent. For instance, lower BMI (<18.5 kg/m^2^) dramatically increased the sarcopenia risk in females instead of males. In addition, the lowest interval of BF% in females also harmed muscle status. Adipose tissue is an essential endocrine organ that regulates hormonal levels. The lowest BMI and BF% resulted in low estrogen levels in menopausal female (42). It was reported that reduction of estradiol concentrations attenuated satellite cell proliferation, and the ability to maintain muscle mass and strength (43). The excess accumulation of fat also affects muscle phenotypes metabolically (17). Hence, we identified a range of BF% to prevent both sarcopenia and obesity in females. Males with the highest interval of BF% had three times greater risk of sarcopenia than the lowest subgroup. In a large cohort of men, increment of fat mass was associated with mortality, which may be associated with the high prevalence of sarcopenia (4). It is necessary to control the adiposity levels in old males due to the faster increasing trend of obesity compared to females (44). BMI was not as sensitive as BF% to simultaneously identify metabolic and sarcopenic risks. From our results, it is recommended for females to have a BMI between 18.5 kg/m^2^ and 25 kg/m^2^, and BF% between 26.0% and 34.6% to prevent sarcopenia and obesity. For males, the BMI should be lower than 25 kg/m^2^ and BF% lower than 23.9%. Those with high BF% warrants early attention due to the higher potential to suffer both muscle and metabolic disorders. Since muscle disorders are associated with high risk of mortality, the reservation of muscle mass and strength is important (9). At present, numerous home-based, economical body fat percentage analysis instruments have been utilized for general body composition supervision, which old people will greatly benefit from. We also recommend that annual health examinations can consider to include BF% in elderlies, and body composition can be maintained through regular exercise and nutrition supplements.

Our study has several strengths. This study exhibited the correlation between various muscle and fat indicators comprehensively. We compared the role of obesity in sarcopenia with two different obesity definitions, and found that higher body fat percentage is related to the increased risk of sarcopenia, but higher BMI is associated with the lower risk of sarcopenia. Our findings indicate that body composition should be focused on in the elderly to observe the risks of both sarcopenia and obesity. The optimal range of BMI and BF% to resist sarcopenia for elderly individuals has also been shown in this study.

There are some limitations in this study. We diagnosed sarcopenia based on the AWGS 2019 consensus with only ASMI and HGS. This is due to the fact that the EWGSOP2 consensus only requires these two parameters for diagnosis, and the addition of physical performance defines severity. We wanted to avoid confusion from readers worldwide. However, as recommended by AWGS 2019 consensus, physical performance parameters such as 6-metre walk, short physical performance battery (SPPB), or 5-time chair stand test should also be evaluated in future studies. In addition, we used a prediction model to estimate ASMI, so that an error from the true value may be present. The sample size of male participants was smaller which may cause the false-negative results. The blood samples as well as comorbidity information were not collected for further analyses. This was a cross-sectional study which only showed the relative risk instead of revealing the causal relationship between obesity and sarcopenia, and thus prospective studies are warranted.

Our study revealed that muscle mass and strength elevated along with BMI and absolute fat mass increment. Obesity is a protective factor of sarcopenia when defined by BMI but is a risk factor when defined by BF%. As for the fat distribution, appendicular fat mass percentage was inversely relevant to muscle mass in both genders, and trunk fat mass percentage was negatively related to muscle strength only in males. The prevalence of SO in Chinese old people was higher if obesity was defined by BF% than BMI. In females with obesity, the annual rate of muscle mass and strength decline was faster than the non-obese group, but this finding did not present in males. The lowest incidence of sarcopenia was found in females with the BF% 26.0–34.6%, and BMI over 18.5 kg/m^2^. A trend showed that BF% less than 23.9% in males was better for sarcopenia prevention. Due to the negative effects of adipose tissue on muscle in pre-clinical studies, a longitudinal obese cohort to explore the alterations of muscle and its function with advanced age is warranted to elucidate the role of fat in muscle clinically.

## Data availability statement

The datasets presented in this article are not readily available unless a valid and reasonable purpose is given. Requests to access the datasets should be directed to louischeung@cuhk.edu.hk.

## Ethics statement

The studies involving human participants were reviewed and approved by The Joint Chinese University of Hong Kong – New Territories East Cluster Clinical Research Ethics Committee (Ref. CREC 2018.602). The patients/participants provided their written informed consent to participate in this study.

## Author contributions

CL: writing-original draft and editing; conceptualization; methodology. KY-KC: investigation; data curation. XT: statistical analysis; data visualization. W-HC: supervision; writing-review and editing. SK-HC: supervision; writing-review and editing. SL: conceptualization; validation. RW: conceptualization; investigation; supervision; writing-review and editing. All authors contributed to the article and approved the submitted version.
