# Supplementary material for: The role of obesity in sarcopenia and the optimal body composition to prevent against sarcopenia and obesity
Source: Front Endocrinol (Lausanne). 2023 Mar 1;14:1077255. doi: 10.3389/fendo.2023.1077255 (PMC10016224; doi:10.3389/fendo.2023.1077255)

Supplementary Material

# Supplementary Table S1. The Regression models of ASMI (kg/m^2^) and HGS (kg) with increased age based on BMI-and BF%-defined obese and non-obese groups.

| Group | Regression model | R^2^ | p-value |
| --- | --- | --- | --- |
| F-O-BMI | y _ASMI_ = -0.017*(Age) + 6.562 | 0.074 | <0.001 |
| F-NO-BMI | y _ASMI_ = -0.006*(Age) + 5.207 | 0.124 | <0.001 |
| F-O-BMI | y _HGS_ = -0.238*(Age) + 35.582 | 0.143 | <0.001 |
| F-NO-BMI | y _HGS_ = -0.206*(Age) + 32.889 | 0.124 | <0.001 |
| F-O-BF% | y _ASMI_ = -0.013*(Age) + 5.944 | 0.032 | <0.001 |
| F-NO-BF% | y _ASMI_ = -0.004*(Age) + 5.170 | 0.004 | 0.05 |
| F-O-BF% | y _HGS_ = -0.253*(Age) + 36.372 | 0.175 | <0.001 |
| F-NO-BF% | y _HGS_ = -0.189*(Age) + 31.818 | 0.101 | <0.001 |
| M-O-BMI | y _ASMI_ = -0.041*(Age) + 9.219 | 0.227 | <0.001 |
| M-NO-BMI | y _ASMI_ = -0.037*(Age) + 8.287 | 0.254 | <0.001 |
| M-O-BMI | y _HGS_ = -0.378*(Age) + 55.042 | 0.167 | <0.001 |
| M-NO-BMI | y _HGS_ = -0.497*(Age) + 63.281 | 0.317 | <0.001 |
| M-O-BF% | y _ASMI_ = -0.041*(Age) + 8.805 | 0.235 | <0.001 |
| M-NO-BF% | y _ASMI_ = -0.045*(Age) + 9.104 | 0.261 | <0.001 |
| M-O-BF% | y _HGS_ = -0.420*(Age) + 56.429 | 0.233 | <0.001 |
| M-NO-BF% | y _HGS_ = -0.461*(Age) + 61.647 | 0.268 | <0.001 |

# Supplementary Table S2. Odds ratio (95% CI) of sarcopenia incidence according to the BMI and BF% distribution.

|  | Male | | | |  | Female | | | |
| --- | --- | --- | --- | --- | --- | --- | --- | --- | --- |
|  | OR | 95% CI | | p-value |  | OR | 95% CI | | p-value |
|  |  | lower | upper |  |  |  | lower | upper |  |
| BMI |  |  |  |  |  |  |  |  |  |
| <18.5 | 1.04 | 0.34 | 3.17 | 0.94 |  | 1.50 | 1.00 | 2.25 | 0.05 |
| 18.5 – 22.9 | 1.00 (reference) | |  |  |  | 1.00 (reference) | |  |  |
| 23 – 24.9 | 0.74 | 0.39 | 1.40 | 0.36 |  | 0.91 | 0.68 | 1.22 | 0.53 |
| 25 – 29.9 | 0.49 | 0.27 | 0.88 | 0.02 |  | 0.84 | 0.64 | 1.10 | 0.21 |
| ≥30 | 0.35 | 0.08 | 1.48 | 0.17 |  | 0.41 | 0.24 | 0.69 | 0.001 |
|  |  |  |  |  |  |  |  |  |  |
| Fifth of BF% |  |  |  |  |  |  |  |  |  |
| 1 (lowest) | 1.00 (reference) | |  |  |  | 1.00 (reference) | |  |  |
| 2 | 0.99 | 0.45 | 2.17 | 0.98 |  | 0.63 | 0.45 | 0.89 | 0.01 |
| 3 | 1.79 | 0.85 | 3.75 | 0.12 |  | 0.67 | 0.48 | 0.94 | 0.02 |
| 4 | 1.79 | 0.85 | 3.75 | 0.12 |  | 0.94 | 0.67 | 1.32 | 0.73 |
| 5 (highest) | 3.26 | 1.51 | 7.07 | 0.002 |  | 1.04 | 0.74 | 1.46 | 0.81 |

**Supplementary Figure S1.**  The correlation between muscle and fat indicators in males (**Supplementary Figure 1A**) and females (**Supplementary Figure 1B**). The dark blue showed the strong positive correlation (correlation coefficient = 1), while the dark red showed the strong negative correlation (correlation coefficient = –1). Black cross was shown if there was no statistical significance (P > 0.05). The correlation coefficient was displayed in the lower half of the square. Abbreviation: AFM%, arm fat mass percentage; LFM%, leg fat mass percentage; BF%, body fat percentage; TFM%, trunk fat mass percentage; BMI, body fat index; WHR, waist to hip ratio; LFM, leg fat mass; FMI, fat mass index; AFM, arm fat mass; BFM, body fat mass; TFM, trunk fat mass; AFFM%, arm fat-free mass percentage; LFFM%, leg fat-free mass percentage; SMI, skeletal muscle mass index; ASMI, appendicular skeletal muscle mass index; SMM, skeletal muscle mass; AFFM, arm fat-free mass; HGS, handgrip strength; LFFM, leg fat-free mass.


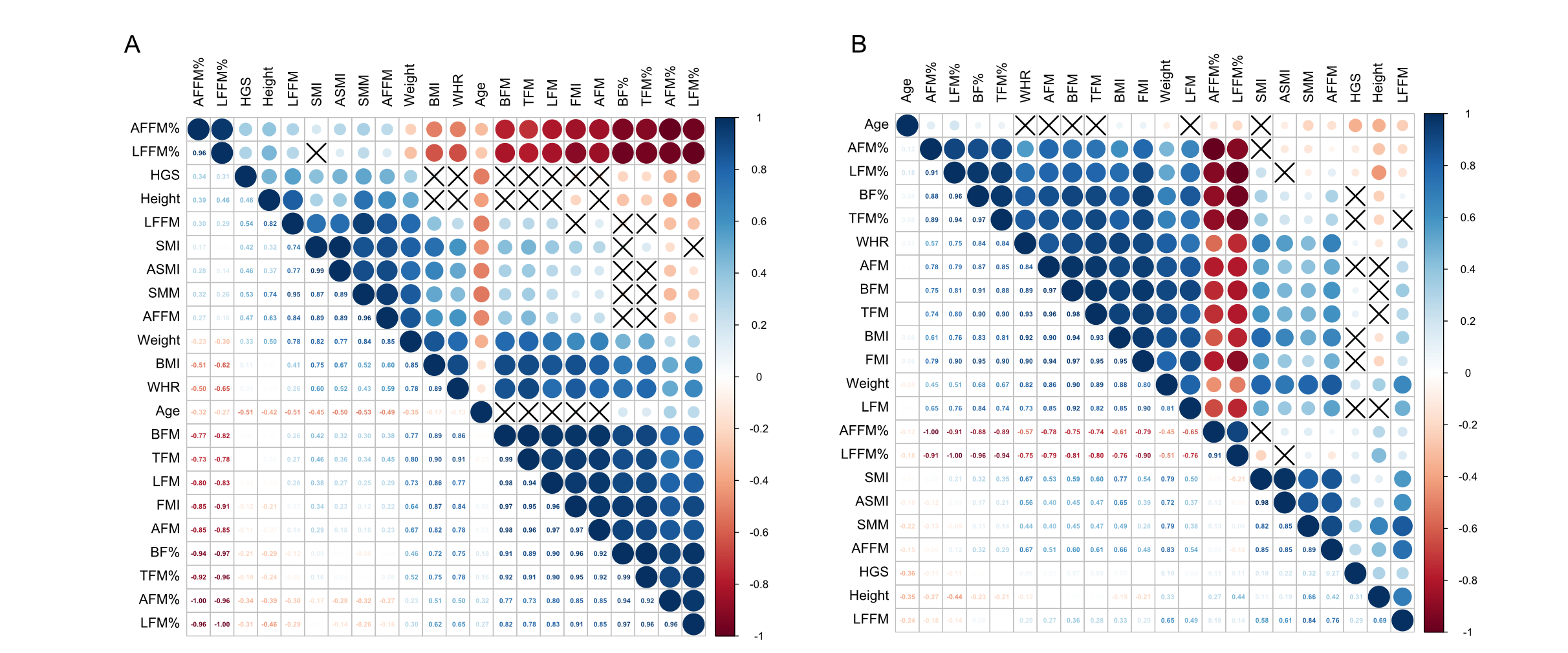

Supplement: Supplementary file 1 [file DataSheet_1.docx]
